# Correlates of home and neighbourhood-based physical activity in UK 3–4-year-old children

**DOI:** 10.1093/eurpub/ckw067

**Published:** 2016-05-11

**Authors:** Jill A. Hnatiuk, Kathryn R. Hesketh, Esther M.F. van Sluijs

**Affiliations:** 1 School of Science and Health, Western Sydney University, Locked Bag 1797, Penrith, NSW, Australia;; 2 UCL Institute of Child Health, London, UK;; 3 MRC Epidemiology Unit and Centre for Diet and Activity Research (CEDAR), Cambridge, UK

## Abstract

**Background:** Identifying context-specific correlates of home- and neighbourhood-based physical activity in preschool-aged children may help improve intervention program development for these settings. **Methods:** A total of 153 3–4-year-old children were recruited through preschool settings in Cambridgeshire (January–July 2013). Children wore Actiheart accelerometers for ≤7 days to assess their sedentary time (ST), light-(LPA) and moderate- to vigorous-intensity physical activity (MVPA). A parent-completed questionnaire assessed correlates across the ecological model and the child’s preschool attendance during the measurement week. Only accelerometer data for times when children were at home were used. Multilevel models (Level 1: days; Level 2: child) examined associations between maternal-reported exposure variables and each outcome (children’s home- and neighbourhood-based ST, LPA and MVPA) (main analysis). Further analyses included the subsample of children with complete paternal correlates data (father analysis). **Results:** In the main analyses, children with older siblings engaged in less ST. Children whose mothers reported being ‘moderately inactive’ or ‘active’ (vs. inactive) engaged in less LPA, while children whose mothers worked >35 h week^−1^ engaged in less MVPA. More equipment at home was associated with lower LPA but greater MVPA. In the father analysis, father’s television viewing before 6 pm was associated with greater ST and less MVPA in children; the negative association between mother’s activity and children’s LPA was retained. **Conclusion:** Family demographics and parental behaviours appear to have the strongest association with children’s home- and neighbourhood-based ST, LPA and MVPA. This study further highlights the importance of examining both maternal and paternal behaviours.

## Introduction

Optimizing physical activity and minimizing sedentary behaviour in the early childhood period (<5 years of age) has become a public health priority in recent years.^1^ Many government organizations internationally now recognize the growing evidence for the importance of these health behaviours with physical activity and sedentary behaviour recommendations for children under the age of 5.^2^ However, population estimates of physical activity and sedentary time (ST) of children at the commencement of primary school suggest that many children are insufficiently active,^3^ and identifying strategies to increase physical activity participation and minimize ST in early childhood is required. This is often done through the implementation of public health interventions.^4^

A key mechanism for informing evidence-based intervention programming is through the investigation of correlates of children’s physical activity and sedentary behaviour.^4^ The examination of correlates of children’s activity behaviour in the early childhood period is a growing area of interest with a considerable number of studies published within the last decade.^5^ Much of this research has focused on correlates of activity behaviours accumulated during the whole day, across multiple settings.^5^ However, behavioural correlates are suggested to be domain-specific,^6^ and focusing on the examination of correlates within specific settings (e.g. home, childcare, community) may provide more targeted direction for intervention programming within these settings.^7^ Although several studies have focused on correlates of preschool children’s physical activity and/or ST in the childcare or preschool setting,^8–11^ there remains a dearth of information regarding context-specific correlates of physical activity outside of formal care.

The home environment has been shown to be an important influence on children’s activity behaviours,^12^ but identifying the time that young children are within the home environment during the day can be difficult. While the majority of children living in developed countries age ≥5 attend primary school,^13^^,^^14^ younger children often have varying care arrangements. For example, they may attend formal childcare full or part time, attend informal childcare regularly (e.g. a childminder or non-registered home-based provider), or attend informal childcare irregularly (e.g. occasional care services, gym crèche, etc.) throughout the week. Here, we use individual-level data and information about children’s care to investigate correlates of young children’s ST, light- (LPA) and moderate- to vigorous-intensity physical activity (MVPA) undertaken in home and neighbourhood settings.

## Methods

### Participants

Data were from the ‘Studying Physical Activity in preschool aged Children and their Environment’ (SPACE) study, a cross-sectional study conducted in 3–4-year-old children and their parents. The details of recruitment are described elsewhere.^15^ In brief, participants were recruited through preschool and nursery centres in the Cambridgeshire area between January and July 2013. Centres were identified from government lists, and were stratified by type (preschool or nursery) and tertile of area deprivation (Index of Multiple Deprivation).^16^ Centres were randomly selected within strata and invited to participate; only those providing centre-level consent were included (*n* = 30; 38% response rate). Ethics approval was granted by the University of Cambridge Psychology Ethics Committee (Pre.2012.68).

All parents of potentially eligible children (*n* = 602) within consenting centres were provided with an information pack and were asked to return a written consent form if they wished for their child to participate. Children were eligible to take part if they: were 3–4-years-old; were registered to attend on the designated measurement day; were free from physical disability; and attended the setting for at least 9 h week^−1^ (to ensure children spent >50% of their government-paid allocation [15 h] at that particular setting). In addition, a minimum of five children per setting with valid written consent was required to ensure sufficient analytical power for the broader study.

### Data collection procedures

Measurements were conducted at centres; children with valid consent but absent on the measurement day were offered a home visit to maximize participation. At the centre visit, Actiheart monitors were fitted to assess children’s free-living activity. The Actiheart device is a combined lightweight heart-rate monitor and accelerometer and has been previously validated for use in preschool children.^17^ The Actiheart monitors were set to record in 15-s epochs and children were encouraged to wear the device continuously (day, night and during water-based activities) for 7 days. During the visit, children’s height was measured to the nearest 0.1 cm using a Leicester stadiometer, and weight to the nearest 0.1 kg using Seca digital scales in light indoor clothes and socks. Parents received a questionnaire, based on a previously validated measure,^18^ which assessed demographic characteristics of the family and a range of potential correlates of children’s physical activity and sedentary behaviour. This questionnaire also included a specially designed question to capture the child’s location during the measurement week.^15^ Questionnaires and Actiheart monitors were collected from centres 1 week later.

### Children’s home-based physical activity

Only accelerometry data were used because combined heart-rate data have been shown to explain little additional variation in estimates of free-living physical activity in pre-schoolers.^19^ Accelerometer data from the Actiheart monitors were downloaded and processed in Stata 13/SE. Actiheart counts were converted to the ActiGraph 7164 equivalent using a conversion factor of five and periods of ≥100 min with zero-activity counts were removed.^20^ All physical activity data captured between the hours of 6 am and 9 pm were processed. Between 9 pm and 11 pm, data were excluded if 45 min in the hour were classified as sedentary,^21^ assuming sleep. Pate et al.^22^ cut-points were used to determine the time spent sedentary (0–37.5 counts per 15 s), in LPA (>37.5- <420 counts per 15 s) and in MVPA (≥420 counts per 15 s). To enable matching to location data, activity data were processed in 15-min epochs aggregated for each 15-min segment and subsequently summed for each hour if four segments were available.

Activity and parent-reported location data were individually matched for every recorded 15-min segment between 6 am and 11 pm. Only segments categorized as ‘at home’ were used in the present analyses; children were considered ‘at home’ if parents reported that the children were with parents (mummy, daddy, us, etc.), grandparents or a nanny, or during any time periods when parents did not specify that their child was in care. In addition, given some children spent a larger proportion of their day in childcare compared with others, children were only included in analyses if they wore the monitor for at least 10 h of time considered ‘at home’ per day over one or more days. This criterion is comparable to what is generally considered a valid full day for research on preschool aged children.^23^ We did not distinguish between weekdays and weekend days as average physical activity levels did not differ between weekdays at home and weekend days.^15^ All physical activity data were divided by the total accelerometer wear time ‘at home’ and multiplied by 60 to generate outcome variables expressed as average minutes per hour (min/h).

### Exposure and confounding variables

A range of correlates across the levels of the social-ecological model^24^ were assessed in the parent questionnaire and through the anthropometric measurements taken (Child’s z-BMI). Context-specific correlates were identified and subsequently grouped into six blocks of correlates using level of the model as a framework: individual, family demographic, parental support, maternal behaviours, paternal behaviours, home environment (see table 1 for a detailed description). In addition to these exposure variables, data on the following confounders were collected: child’s sex (male/female), maternal and paternal education (low = General Certificate of Secondary Education, Advanced Level, National Vocational Qualification & Diploma; medium = university degree; high = higher degree), maternal and paternal age, and season (winter [January–February]; spring [March–May]; summer [June–July]). The total time in care was calculated by summing the reported hours ‘in care’ as described previously.

**Table 1:** Description of correlates of children’s physical activity and ST examined by block

Variable name	Description and/or coding
Block 1: Individual correlates	
Child z-BMI	Calculated using the LMS method.^25^ IOTF cut-off scores separated children into three categories: healthy, overweight and obese
Child TV time	Five categories of TV time per day (<30 min; 30–<60 min; 1 to < 2 h; ≥2 h)
Child age (months)	Computed using the child’s date of birth and date of measurement visit
Block 2: Family situation correlates	
Maternal age (years)	Computed using the mother’s date of birth and date of the child’s measurement visit
Younger siblings in home	Determined by one item asking the number of children in the home in five age brackets (0–2; 3–5; 6–11; 12–16; 17–18). Younger siblings categorized as yes if parent responded there was a 0–2-year-old child in the home
Similar aged siblings in home	Determined by one item asking the number of children in the home in five age brackets (0–2; 3–5; 6–11; 12–16; 17–18). Younger siblings categorized as yes if parent responded there was another 3–5-year-old child in the home
Older siblings in home	Determined by one item asking the number of children in the home in five age brackets (0–2; 3–5; 6–11; 12–16; 17–18). Older siblings categorized as yes if parent responded there was a child >5 years old living in the home
Maternal BMI	Mother’s height and weight were self-reported. Categorized according to WHO classifications: Healthy: BMI <25 kg m^−2^; Overweight 25 to < 30 kg m^−2^; Obese ≥30 kg m^−2^
Maternal employment	Due to distribution of the data, categorized into: Not employed; <20 h week^−1^; 21–35 h week^−1^; >35 h week^−1^
Paternal age^a^	Computed using the father’s date of birth and date of the child’s measurement visit
Paternal BMI^a^	Father’s height and weight was self-reported. Categorized according to WHO classifications: Healthy: BMI <25 kg m^−2^; Overweight 25 to < 30 kg m^−2^; Obese ≥30 kg m^−2^
Paternal employment^a^	Due to distribution of the data, categorized into: <40 h week^−1^; 40–42 h week^−1^; >42 h week^−1^
Block 3: Parental support correlates	
Parent encouragement	Composite score calculated as the mean of two items: frequency of doing physical activity with the child and encouraging physical activity (1 = never; 5 = very often)^18^
Parent logistic support	Composite score calculated as the mean of two items: frequency of transporting child to physical activities and watching the child do physical activity (1 = never; 5 = very often)^18^
Parent modelling	Composite score calculated as the mean of four items assessing the frequency child sees parents doing physical activity (1 = never; 5 = very often)^26^
Block 4: Maternal behaviour correlates	
Short travel mode	Parents reported their usual travel mode for short trips (<1/2 mile): categorized as: parent and child active; parent active child inactive; both parent and child inactive^18^
Maternal TV (before 6 pm)	Composite weighted score of weekday and weekend television viewing before 6 pm. Individual items had six response options (None; <1 h day^−1^, 1–2 h day^−1^; 2–3 h day^−1^; 3–4 h day^−1^; >4 h day^−1^)^27^
Maternal TV (after 6 pm)	Composite weighted score of weekday and weekend television viewing after 6 pm. Individual items had six response options (None; <1 h day^−1^, 1–2 h day^−1^; 2–3 h day^−1^; 3–4 h day^−1^; >4 h day^−1^)^27^
Maternal computer use (before 6 pm)	Composite score of weekday and weekend computer use before 6 pm. Individual items had six response options (None; <1 h day^−1^, 1–2 h day^−1^; 2–3 h day^−1^; 3–4 h day^−1^; >4 h day^−1^)^27^
Maternal computer use (after 6 pm)	Composite score of weekday and weekend computer use after 6 pm. Individual items had six response options (None; <1 h day^−1^, 1–2 h day^−1^; 2–3 h day^−1^; 3–4 h day^−1^; >4 h day^−1^)^27^
Maternal leisure time physical activity	Previously validated index of leisure time physical activity.^28^ 0 h week^−1^ = inactive; 0.1–3.5 h week^−1^ = moderately inactive; 3.6–7.0 h week^−1^ = moderately active; >7.0 h week^−1^ = active
Block 5: Home environment correlates	
Space in home	Number of locations in home conducive to physical activity (e.g. yard, inside playroom, driveway, etc.) selected (range 1–6). Adapted from Gattshall et al.^26^
Equipment in home	Number of physical activity equipment items appropriate for young children in home (range 1–9). Adapted from Gattshall et al.^26^
Equipment accessibility	Composite score of four items assessing the ability of children to access and use the equipment in home (1 = None; 5 = All). Adapted from Gattshall et al.^26^
Stranger concerns	One item assessing parental concerns about stranger danger. Scored on 5-point scale collapsed into: 1 = strongly disagree/disagree; 2 = neither; 3 = agree/strongly agree^18^
Traffic concerns	One item assessing parental concerns about road safety. Scored on 5-point scale collapsed into: 1 = strongly disagree/disagree; 2 = neither; 3 = agree/strongly agree^18^
Block 6: Paternal behaviour correlates^a^	
Paternal TV (before 6 pm)	Composite score of weekday and weekend television viewing before 6pm. Individual items had six response options (None; <1 h day^−1^, 1–2 h day^−1^; 2–3 h day^−1^; 3–4 h day^−1^; >4 h day^−1^)^27^
Paternal TV (after 6 pm)	Composite score of weekday and weekend television viewing after 6pm. Individual items had six response options (None; <1hr/day, 1-2 hrs/day; 2-3 hrs/day; 3-4 hrs/day; 4+ hrs/day)^27^
Paternal computer use (before 6 pm)	Composite score of weekday and weekend computer use before 6 pm. Individual items had six response options (None; <1 h day^−1^, 1–2 h day^−1^; 2–3 h day^−1^; 3–4 h day^−1^; >4 h day^−1^)^27^
Paternal computer use (after 6 pm)	Composite score of weekday and weekend computer use after 6 pm. Individual items had six response options (None; <1 h day^−1^, 1–2 h day^−1^; 2–3 h day^−1^; 3–4 h day^−1^; >4 h day^−1^)^27^
Paternal leisure time physical activity	Previously validated index of leisure time physical activity.^28^ 0 h week^−1^ = inactive; 0.1–3.5 h week^−1^ = moderately inactive; 3.6–7.0 h week^−1^ = moderately active; >7.0 h week^−1^ = active

^a^Only assessed in the secondary analyses using a sub-sample of children with complete maternal and paternal data.

### Data analysis

All analyses were conducted using STATA 13/SE. Proportions and means were derived as descriptive statistics. Comparisons between those included in analyses and those excluded were examined using *t*-tests and Pearson’s *χ*^2^. Multilevel linear regression (Level 1: days; Level 2: child) was used to examine associations between exposure variables and the three outcomes (min/h spent sedentary and in LPA and MVPA). As previous research has shown differences in correlates for boys and girls,^29^ interactions by sex were explored for one randomly selected variable in each of the six blocks. As no significant interactions (at *P* < 0.05) were observed, analyses were run with boys and girls combined.

A three-stage analysis strategy was applied. First, to determine the influence of ecological level (individual, family demographic, parental support, maternal behaviours, paternal behaviours, home environment), associations between each block and the outcome variables were examined independently, controlling for total time in care, child’s sex and maternal education. Each block was then tested separately against the null model (which comprised only confounding variables) using a likelihood ratio (LR) test. Blocks providing a better fit over the null model (*P* < 0.10) were retained. Second, individual correlates out of the retained blocks showing a statistically significant association with the outcome in simple models (*P* < 0.05) (controlling for confounders) were taken forward to a multivariable model. Third, a multivariable model was run including all significant individual exposure variables from all retained blocks, controlling for confounders.

This analytical strategy was used for each of the three outcome variables (LPA, MVPA and ST), initially on the full sample of children with maternal behavioural data (*n* = 153), and subsequently on the sub-sample of children with complete paternal behavioural data (*n* = 120). These additional analyses were performed to examine the association between paternal correlates, in the context of maternal factors, with children’s physical activity and ST.

## Results

### Participants

Of the 234 children who were fitted with Actiheart monitors and given parental questionnaires, 32 had insufficient physical activity data (<10 h of valid physical activity data ‘at home’) and a further 49 had incomplete questionnaire data. This left 153 children for inclusion in the final analyses. Table 2 shows the participant characteristics of this sample. Mothers of children included were slightly younger (36.9 vs. 37.5 years; *P* < 0.05) and were less likely to have a higher degree compared with those excluded from analyses (*χ*^2^ = 9.75, *P* < 0.05). No differences were observed between groups for maternal BMI.

**Table 2:** Demographic characteristics and physical activity levels of participants included in analyses (*n* = 153)

Characteristic	
Children	
Sex of child (% male)	49.4%
Hours per day of monitor wear ‘at home’	14.1 (1.1)
Days of monitor wear ‘at home’ [mean (SD)]	4.2 (1.5)
Minutes per hour spent sedentary [mean (SD)]	22.4 (5.8)
Minutes per hour spent in light-intensity physical activity [mean (SD)]	22.8 (3.4)
Minutes per hour spent in MVPA [mean (SD)]	14.9 (6.6)
Parents	
Maternal age in years [mean (SD)]	37.5 (5.1)
Maternal education (%)	
Low (Secondary school or diploma)^a^	30.7%
Mid: (Bachelor’s degree)	32.7%
High: (Higher degree)	36.6%
Paternal age in years (mean (SD))^a^	39.7 (7.0)
Paternal education (%)^b^	
Low (Secondary school or diploma)^a^	23.1%
Mid: (Bachelor’s degree)	27.3%
High: (Higher degree)	49.6%

^a^General Certificate of Secondary Education, Advanced Level, or National Vocational Qualification.

^b^Paternal sample: *n* = 120.

### Correlates analyses

For children’s ST, only the ‘family demographics’ block provided a better fit over the null model (LR *χ*^2^ = 17.50, *P* < 0.04). For children’s LPA, two blocks provided a better fit over the null model (‘maternal behaviours’: LR *χ*^2 ^= 15.14, *P* < 0.08 and ‘home environment’ LR *χ*^2 ^= 12.96, *P* < 0.07, respectively). For children’s MVPA, the ‘family demographics’ (LR *χ*^2 ^= 14.97, *P* < 0.09) and ‘home environment’ (LR *χ*^2 ^= 14.51, *P* < 0.04) blocks provided a better fit over the null model. Table 3 outlines the results of the final multivariable models, in which only those individual correlates that showed a statistically significant association in simple models were retained. Children with older siblings spent less time sedentary (*β* = −2.32, 95%CI [−4.29; −0.34]). Compared with children whose mothers were considered ‘inactive’, those whose mothers reported being ‘moderately inactive’ (*β* = −1.63, −3.14; −0.13) or ‘active’ (*β* = −2.15, −4.32; −0.07) engaged in less LPA, while children whose mothers worked >35 h week^−1^ engaged in less MVPA (*β* = −3.37, −6.38; −0.36). More equipment at home was associated with lower LPA (*β* = −0.39, −0.73; −0.04) but greater MVPA (*β* = 13.34, 8.40; 18.38).

**Table 3:** Multivariate associations between significant correlates within blocks and children’s SED, LPA and MVPA (minutes per hour) (*n* = 153)^a^

	SED β (95% CI)	LPA β (95% CI)	MVPA β (95% CI)
Family situation			
Any younger siblings	−2.02 (−4.13, 0.79)	–	–
Any older siblings	−**2.32 (**−**4.29,** −**0.34)**	–	1.35 (−0.79, 3.49)
Maternal employment			
Not employed	Ref.	–	Ref
<20 h week^−1^	−0.69 (−3.38, 1.99)	–	−0.23 (−3.32, 2.85)
21–35 h week^−1^	−0.49 (−2.68, 1.71)	–	−0.93 (−3.43, 1.57)
>35 h week^−1^	**2.93 (0.34, 5.54)**	**–**	−**3.37 (**−**6.38,** −**0.36)**
Maternal behaviours			
Maternal computer use before 6pm	–	−0.70 (−1.39, 0.03)	–
Mother’s physical activity			
Inactive	–	Ref.	–
Moderately inactive	**–**	−**1.63 (**−**3.14,** −**0.13)**	–
Moderately active	–	−1.67 (−3.42, 0.07)	–
Active	**–**	−**2.15 (**−**4.23,** −**0.07)**	–
Home environment			
Equipment in home	**–**	−**0.39 (**−**0.73,** −**0.04)**	**13.34 (8.40, 18.38)**

^a^Adjusted for time in care, child’s sex, maternal education and season; bold indicates significance at p < 0.05

– Not assessed in the analysis for the respective outcome variable.

For the father analysis (*n* = 120), the ‘paternal behaviours’ block improved the model fit over the null model for both ST (LR *χ*^2 ^=24.41, *P* < 0.08) and MVPA (LR *χ*^2 ^= 24.31, *P* < 0.08). Table 4 shows the results of the final multivariable models in this reduced sample with paternal data. Most notably, greater paternal TV viewing before 6 pm was associated with higher ST (*β* = 2.36, 0.40; 4.33) and lower MVPA (*β* = −2.45, −4.49; −0.42). Furthermore, the inclusion of paternal data strongly attenuated the association with equipment in the home, maternal employment and older siblings.

**Table 4:** Multivariate associations between correlates and children’s SED, LPA and MVPA (minutes per hour) in the sub-sample of children with both maternal and paternal data (*n* = 120)

	SED β (95% CI)	LPA β (95% CI)	MVPA β (95% CI)
Family situation			
Any younger siblings	−2.03 (−4.38, 0.32)	–	–
Any older siblings	−1.96 (−4.23, 0.32)	–	1.74 (−0.61, 4.10)
Maternal employment			
Not employed	Ref.	–	Ref.
<20 h week^−1^	0.47 (−2.52, 3.46)	–	−1.44 (−4.75, 1.87)
21–35 h week^−1^	−0.02 (−2.46, 2.41)	–	−1.40 (−4.06, 1.26)
>35 h week^−1^	2.66 (−0.47, 5.79)	–	−2.75 (−6.09, 0.59)
Maternal behaviours			
Maternal computer use before 6pm	–	−0.64 (−1.49, 0.21)	–
Mother’s physical activity			
Inactive	–	Ref.	–
Moderately inactive	**–**	−**2.42 (**−**4.10,** −**0.74)**	–
Moderately active	–	−1.43 (−3.35, 0.50)	–
Active	**–**	−**2.91 (**−**5.23,** −**0.59)**	–
Paternal behaviours			
Paternal TV before 6 pm	**2.36 (0.40, 4.33)**	**–**	−**2.45 (**−**4.49,** −**0.42)**
Paternal TV after 6 pm	−0.60 (−1.70, 0.51)	–	–
Home environment			
Equipment in home	–	−0.36 (−0.74, 0.02)	0.71 (−0.34, 1.46)

^a^Adjusted for time in care, child’s sex, maternal education and season; bold indicates significant at 0.05.

–Not assessed in the analysis for the respective outcome variable.

## Discussion

This study is one of the first to examine correlates of preschool-aged children’s home- and neighbourhood-based activity behaviour. Our findings from the main analyses are similar to some previous works in other preschool-aged populations. Earlier studies have found that the presence of older siblings in the household was positively associated with children’s MVPA^30^ and total physical activity^29^ and having siblings of any age was associated with less television viewing time^31^ and greater MVPA.^32^ This study extends these findings to include objectively-measured ST. In addition, contrary to previous work,^5^^,^^33^ we found a negative relationship between maternal employment and children’s MVPA. However, both these findings were attenuated with the addition of paternal correlates into the model. Although the sample size was reduced in the father analyses, re-analyses of the main analysis in the smaller sample (*n* = 120) did not show a major impact of sample size on the conclusions (results not shown). This suggests that having older siblings and maternal employment are not uniquely associated with children’s physical activity and ST when considered alongside other relevant family correlates.

In the main analyses, more equipment in the home was associated with greater MVPA and less LPA in children. This finding suggests that equipment availability may enable children to replace some of their LPA with MVPA. This is consistent with research in the preschool environment whereby portable play equipment has been positively associated with children’s MVPA.^34^ Given recommendations suggest preschool children should be working towards accumulating at least 60 min of MVPA by age 5,^35^ provision of equipment may be a useful strategy to enable higher intensity activity. It is not clear whether this similarly influences time spent sedentary. Moreover, as with maternal employment and the presence of older siblings, when the paternal correlates were added, this association was attenuated. Thus, it may not necessarily be the equipment itself that is associated with children’s MVPA, but that the equipment availability in the home is reflective of a parent (in this case, father) who is more likely to engage in active play with their child during the day rather than engage in more sedentary pursuits.

Maternal self-reported physical activity was negatively associated with children’s LPA. This is in contrast to most,^29^^,^^30^^,^^36^ but not all,^37^ studies using objectively-measured maternal activity. The self-report measure used here assessed activity across multiple domains, including leisure time and transport-related, and therefore, it is likely to have captured a broader range of physical activities than those mothers engaged in when with their child. Given maternal activity remained significant in the father analysis, further research into the specific relationship between maternal and child activity is warranted.

The father analysis showed that greater paternal television viewing time before 6 pm was associated with reduced ST and greater MVPA amongst children at home. This indicates that fathers’ health behaviours, in particular their daytime television viewing, may be an important, independent influence on preschool children’s physical activity and ST, over and above maternal correlates and the home environment. This finding is also consistent with other observational research in preschool children^38^ and experimental research in primary school children^39^ and highlights the importance of collecting data from both parents in two-parent families. This study may also suggest that all screen viewing by fathers may not be equal; that is, paternal daytime television viewing may have a greater impact on children’s behaviour compared with that viewed in the evening periods. This is similar to findings which suggest that maternal-child co-participation in sedentary behaviour is associated with lower physical activity in 1–3-year-old children during the morning and afternoon, but not the evening.^37^ It is possible that paternal television viewing during the daytime when children are awake results in higher co-participation in this behaviour together, though it is not possible to determine this from this study. Future studies may therefore wish to consider examining family members’ activity and screen behaviours during the daytime and evening. If consistent findings emerge, this could be a tangible recommendation (e.g. limiting screen during daytime hours) for public health professionals working with young families and intervention programs delivered within the community.

Broadly, the findings from this study suggest that the social level of the social-ecological model may have the greatest influence on young children’s home and neighbourhood-based physical activity. This is also consistent with other work which has assessed a broad range of correlates across the ecological model whereby a greater number of social level correlates were associated with children’s physical activity compared with individual or environmental level correlates.^29^ Therefore, including a strong focus on social correlates (e.g. people around the child) and considering family demographic characteristics in the developmental of family-based interventions may be vital for optimizing preschool children’s physical activity and minimizing ST in the home environment.

### Strengths and limitations

A key strength of this study is its unique approach in examining correlates of preschool children’s objectively measured physical activity and ST, specifically within home and neighbourhood settings. This is particularly relevant given the varying care arrangements of children of this age. This study examined a range of correlates across all levels of the ecological model, taking into account individual level fluctuations of behaviour using multi-level models. However, the sample size was relatively small, potentially limiting the power to detect smaller associations observed in previous work, and parents were more highly educated than the general UK population, see Hesketh et al.^15^ for further discussion on this issue. In addition, only a few aspects of the neighbourhood environment were assessed and all were based on parent-report. Although attempts were made to minimize the number of tests conducted by first examining associations by level of the ecological model rather than individual correlates, there is a possibility that some findings may have occurred by chance due to the number of statistical tests conducted in this study. Finally, it should be acknowledged that parents who completed the questionnaire may be more involved in the child’s life generally and therefore the same findings may not apply for those whose parents are less engaged.

## Conclusion

This study found that family demographic and parental behaviours have the strongest association with children’s ST, LPA and MVPA in the home and neighbourhood setting. A focus on modifying these factors in future intervention programmes that aim to increase physical activity in home and community settings may increase program efficacy. Furthermore, ensuring that both maternal and paternal data is captured in two-parent families is necessary to better understand correlates of children’s physical activity and sedentary behaviours.

## Supplementary Material

Supplementary Data
