# Supplementary material for: Correlates of home and neighbourhood-based physical activity in UK 3–4-year-old children
Source: Eur J Public Health. 2016 May 11;26(6):947–53. doi: 10.1093/eurpub/ckw067 (PMC5172487; doi:10.1093/eurpub/ckw067)
Supplement: Supplementary Data [file supp_ckw067_ckw067.DC1.html]

Supplementary Data | European Journal of Public Health

## Supplementary Data

files

- Supplementary Data - docx file
