# Supplementary material for: Correlates of home and neighbourhood-based physical activity in UK 3–4-year-old children
Source: Eur J Public Health. 2016 May 11;26(6):947–53. doi: 10.1093/eurpub/ckw067 (PMC5172487; doi:10.1093/eurpub/ckw067)
Supplement: Supplementary Data [file 64e1caa920db2128fa47d1a5d33cba64_ejph-2015-11-om-0835-File002.docx]

**Supplementary Material (online only)**

Likelihood ratio test of significance against base model^a,b^

|  | Block | Likelihood ratio chi^2^ (p-value) |
| --- | --- | --- |
| SED | Individual | 8.46 (0.21) |
|  | Family situation | **17.50 (0.04)** |
|  | Parental support | 1.17 (0.76) |
|  | Maternal behaviours | 9.32 (0.41) |
|  | Home environment | 9.68 (0.21) |
| LPA | Individual | 1.82 (0.94) |
|  | Family situation | 11.70 (0.23) |
|  | Parental support | 2.63 (0.45) |
|  | Maternal behaviours | **15.14 (0.08)** |
|  | Home environment | **12.96 (0.07)** |
| MVPA | Individual | 5.86 (0.44) |
|  | Family situation | **14.97 (0.09)** |
|  | Parental support | 2.73 (0.44) |
|  | Maternal behaviours | 12.80 (0.17) |
|  | Home environment | **14.51 (0.04)** |

**Bolded** values significant at p<0.10

^a^Base model controlled for total time in care, child’s sex, maternal education

^b^Data presented for the full sample, excluding paternal variables (n=153)
